# An exploration of the relationships of experiential avoidance (as measured by the aaq-ii and meaq) with negative affect, perceived stress, and avoidant coping styles

**DOI:** 10.7717/peerj.11033

**Published:** 2021-03-05

**Authors:** M. Todd Allen

**Affiliations:** School of Psychological Sciences, University of Northern Colorado, Greeley, CO, USA

**Keywords:** Avoidance, Experiential avoidance, Coping styles, Negative affect, Perceived stress

## Abstract

**Background:**

Current psychotherapies seek to reduce experiential avoidance (EA) which has also been put forth as a risk factor for anxiety disorders, depression, and post-traumatic stress disorder. EA is a potentially maladaptive self-regulatory tendency to avoid negative thoughts, feelings, memories, physical sensations, and other internal experiences. One unresolved issue with the most commonly used measures of EA, the Acceptance and Action Questionnaire-II (AAQ-II) which measures EA as a single factor and the Multidimensional Experiential Avoidance Questionnaire (MEAQ) which measures EA as six subdimensions, is what exactly is being measured. The AAQ-II appears to measure negative affect (NA), some aspects of avoidant coping, and psychological distress. In addition, the relationships of all the MEAQ subscales have not been thoroughly examined with these other constructs. In the current study, the relationships of AAQ-II and MEAQ scores with NA, avoidant coping styles, and perceived stress were examined.

**Methods:**

Two-hundred undergraduates (154 females and 46 males) completed the AAQ-II and MEAQ, the Distressed Type D Personality Scale (DS-14) which includes a measure of NA, the Brief COPE which measures coping styles, and the Perceived Stress Scale.

**Results:**

Scores on the AAQ-II had moderate positive relationships with the MEAQ total score and all MEAQ subscales with the exception of distress endurance which had a moderate negative relationship. The AAQ-II had a stronger relationship with NA, avoidant coping, and perceived stress than did the MEAQ. All MEAQ subscales had a positive relationship to NA, avoidant coping, and perceived stress with the exception of distress endurance which had a negative relationship with these constructs. While the AAQ-II is limited as a unitary measure of EA the multiple dimensions of the MEAQ may involve an extraneous factor of distress endurance. Future work should examine the relationships of the MEAQ with NA, avoidant coping and perceived stress with clinical populations.

## Introduction

The issue of maladaptive avoidance is addressed in several types of psychotherapy and cognitive–behavioral interventions (e.g., ACT; acceptance and commitment therapy) that are typically designed to help clients reduce avoidant behaviors, reactions, and thoughts (Abramowitz, Deacon & Whiteside, 2013; Dobson & Dobson, 2017) by targeting experiential avoidance or EA (Tyndall et al., 2019). EA is defined as attempts to avoid aversive thoughts, feelings, memories, physical sensations as well as avoiding aversive events or stimuli that produce these internal experiences (Hayes et al., 1996). The most widely utilized measure of EA is the Acceptance and Action Questionnaire-II (AAQ-II; Bond et al., 2011) which measures EA as a unitary construct. The criticism of whether EA should be viewed a single factor or as a multi-faceted construct including cognitive, affective, and behavioral avoidance (Chawla & Ostafin, 2007; Gamez et al., 2011) led to the development of the Multidimensional Experiential Avoidance Questionnaire (MEAQ; Gamez et al., 2011), which includes six subscales assessing behavioral avoidance, distraction and suppression, distress aversion, distress endurance, procrastination, and repression and denial. Gamez et al. (2011) reported that the AAQ-II was positively related to all of the MEAQ subscales with the exception of distress endurance which had a moderate negative relationship. It has been concluded that the MEAQ and AAQ-II do not measure the same construct given that they have only a moderate positive relationship (Lewis & Naugle, 2017; Rochefort, Baldwin & Chmielewski, 2018).

The AAQ-II has also been criticized for questionable discriminative ability in relation to negative affectivity or NA (Gamez et al., 2011; Wolgast, 2014) which is defined as negative emotions including dysphoria, sadness, and a gloomy outlook on life (Denollet, 2005). The MEAQ and its subscales also have a moderate positive relationship with NA with the exception of distress endurance which had a negative relationship (Gamez et al., 2011). More recently, Rochefort, Baldwin & Chmielewski (2018) reported that the NA is more related to the AAQ-II than the MEAQ. The current study sought to replicate these findings with a more recent measure that includes a subscale for NA, the distressed (Type D) personality scale (DS-14; Denollet, 2005).

Another criticism of EA is its relationship to avoidant coping styles. Karekla & Panayiotou (2011) found that higher levels of EA were related to emotion-focused and avoidant coping styles (i.e., self-distraction, denial, emotional support, behavioral disengagement, venting, and self-blame) as measured by the Brief COPE (Carver, 1997). These coping styles have been labeled as maladaptive or toxic (Aldwin & Revenson, 1987; Folkman et al., 1986; Stanton et al., 2000a, 2000b; Kashdan et al., 2006). Denial as measured by the COPE (Carver, Scheier & Weintraub, 1989) was found to have a moderate positive relationship to scores on the MEAQ. To the knowledge of the author, the relationships between all of the MEAQ subscales and all coping styles of the Brief COPE have yet to be examined. Thus, the inclusion of an analysis of these subscales for coping styles and the MEAQ would further our understanding of these relationships.

The AAQ-II has also been criticized for not making a clear enough distinction between the process of EA and the outcome of psychological distress (Chawla & Ostafin, 2007; Wolgast, 2014; Tyndall et al., 2019). Karekla & Panayiotou (2011) found that higher EA as measured by the AAQ-II was associated with higher perceived stress as measured by the perceived stress scale (PSS; Cohen, Kamarck & Mermelstein, 1983). To the knowledge of the author, the relationships between the MEAQ and PSS have not been examined to date.

There has also been no consensus for a gender effect for EA (Bond et al., 2011; Karekla & Michaelides, 2016). Whereas, Panayiotou, Karekla & Leonidou (2017) reported that females women have higher EA (as measured by the AAQ-II) than males, there is no corresponding evidence for a gender effect with the MEAQ (Gamez et al., 2014). Panayiotou, Karekla & Leonidou (2017) also reported that avoidance coping predicts anxiety in women, but not men. However, a recent study found no gender effect on avoidant or non-avoidant coping styles as measured by the Brief COPE (Allen & Myers, 2019). Females are also reported to have higher levels of perceived stress than males (Cohen & Williamson, 1988; Hewitt, Flett & Mosher, 1992; Martin, Kazarian & Breiter, 1995; Karekla & Panayiotou, 2011; Lavoie & Douglas, 2012). Therefore, an examination of the role of gender in EA, negative affect, avoidance coping, and perceived stress was included in the current study.

### Hypotheses

As can be seen from this brief review, several open issues remain for the AAQ-II and MEAQ as measures of EA. It was hypothesized that the AAQ-II would have a moderate positive relationship with the MEAQ total score as well as the MEAQ subscale scores. It was also hypothesized that NA should have a stronger relationship with the AAQ-II than the MEAQ. AAQ-II scores as well as the MEAQ total score and subscales were hypothesized to be positively correlated to the avoidant coping styles and negatively correlated to the non-avoidant coping styles. Scores on the AAQ-II and MEAQ were also hypothesized to be positively correlated to perceived stress. Based on the findings of females having higher AAQ-II scores than males, it was hypothesized that this same effect would be evident with the MEAQ as well as negative affect, avoidant coping, and perceived stress.

## Materials and Methods

### Participants

Two-hundred undergraduates including 154 females and 46 males with a mean age of 19.2 years (SD 2.8, range 18–47) and a mean education level of 13.0 years (SD = 1.2, range 12–17) voluntarily completed the study for partial research participation credit for an introductory psychology course. The ethnicity/racial identity of the sample was mainly Caucasian (*n* = 127) followed by Hispanic (*n* = 23), multi-racial (*n* = 9), African American (*n* = 7), east Asian (*n* = 1) and other (*n* = 1).

### Instruments

The AAQ-II (Bond et al., 2011) is a 7-item self-report measure of EA. It is designed to measure a person’s ability to remain in contact with painful and negative private events (e.g., “I’m afraid of my feelings”; “My painful memories prevent me from having a fulfilling life”). Items are rated on a 7-point Likert-like scale and summed to produce a total score. Internal consistency measured as Cronbach’s alpha is reported to range from 0.76 to 0.87 (Bond et al., 2011; Iverson et al., 2012). Three confirmatory factor analyses have suggested that the AAQ-II involves a single factor (Bond et al., 2011).

The MEAQ (Gamez et al., 2011) is a 62 item self-report inventory consisting of six dimensions including, behavioral avoidance (11 items), distress aversion (13 items), procrastination (seven items), distraction and suppression (seven items), repression and denial (13 items), and distress endurance (11 items). Items are rated on a 6-point Likert-like scale ranging from one (strongly disagree) to six (strongly agree). The MEAQ has been reported to have good psychometric properties including Cronbach’s alpha for individual subscales ranging from 0.70 to 0.85 for repression and denial to behavioral avoidance, respectively (Gamez et al., 2011; Sahdra et al., 2016).

The Distressed Type D Personality Scale (DS-14; Denollet, 2005) includes seven items assessing social inhibition (SI) and seven items assessing negative affect (NA). Items are rated by the participant as to whether it is true or false for him/her on a range of five options including false (zero points), rather false (one points), neutral (two points), rather true (three points) and true (four points). Participants were classified as Type D if they scored 10 or more points on both the SI and NA subscales (Emons, Meijer & Denollet, 2007). The DS-14 has strong psychometric properties including an overall internal consistency with a Cronbach’s alpha of 0.88 for the NA subscale and 0.86 for the SI subscale as well as test retest reliabilities of 0.72 and 0.82 for the NA and SI subscales, respectively (Denollet, 2005).

The Brief COPE (Carver, 1997) is a 28-item questionnaire that includes 14 subscales for acceptance, active coping, behavioral disengagement, denial, emotional support, humor, instrumental support, positive reframing, religion, self-blame, self-distraction, substance use, and venting. These coping styles have been grouped into an avoidance factor consisting of behavioral disengagement, denial, self-blame, self-distraction, and substance use (Baumstarck et al., 2017) while the remaining nine coping styles were grouped as a non-avoidance factor. The Brief COPE has satisfactory test-retest reliability and convergent and divergent validity (Muller & Spitz, 2003; Cooper, Katona & Livingston, 2008). In addition to the overall inventory, the two items for each subscale (Carver, 1997; Muller & Spitz, 2003; Cooper, Katona & Livingston, 2008; Yusoff, 2010) as well as the five subscales forming the aggregate avoidance score (Baumstarck et al., 2017) have acceptable internal reliability.

The PSS (Cohen, Kamarck & Mermelstein, 1983) includes ten items which ask about how often the participant felt and thought a certain way during the last month. Specifically, the PSS assesses “the degree to which individuals appraise situations in their lives as stressful” (Cohen, Kamarck & Mermelstein, 1983; p. 385). Cronbach’s alpha for the 10-item version of the PSS ranged from 0.78 to 0.92 (Cole, 1999; Karekla & Panayiotou, 2011; Lee, 2012).

### Procedure

All participants provided written informed consent at the start of the experiment. They completed a demographic questionnaire that asked about gender, age, and years of education. All procedures were approved by the Institutional Review Board of the University of Northern Colorado (approval #799609-4).

### Data analysis

All scoring was done according to published inventory instructions. The Statistical Package for the Social Sciences (Version 26, IBM Corporation, Armonk, NY, USA) was used for all statistical analyses. Gender differences were tested with two-tailed independent measures *t* tests. Based on the normality of the inventory scores and the large sample size, relationships between inventories were tested with a Pearson product moment correlation. Stepwise linear regressions were computed with predictors of NA, avoidance coping, MEAQ total scores and AAQ-II scores. Dependent variables were scores on the AAQ-II, MEAQ and PSS.

## Results

The overall mean scores and means for males and females for the various inventories are shown in Table 1. The only significant gender effects revealed were that females had higher scores than males on the AAQ-II (*t*(198) = 3.08, *p* = 0.003), the MEAQ subscale for distraction and suppression (*t*(198) = 2.38, *p* = 0.02) and the NA (*t*(198) = 2.26, *p* = 0.02) while males had higher scores than females for distress endurance *t*(198) = 2.08, *p* = 0.04).

**Table 1:** Gender effects for inventories.

	Overall mean (SD)	Female mean (SD)	Male mean (SD)	Significance level
AAQ-II	24.0 (9.5)	25.1 (9.4)	20.3 (9.3)	0.003
MEAQ	208.6 (29.0)	210.6 (30.6)	201.9 (21.6)	ns
Behavioral avoidance	36.1 (7.3)	36.4 (7.6)	34.7 (5.8)	ns
Distress aversion	45.8 (9.2)	46.4 (9.8)	43.7 (6.3)	ns
Procrastination	27.7 (4.3)	27.8 (4.4)	27.5 (4.1)	ns
Distraction & suppression	27.4 (5.2)	27.9 (5.6)	25.8 (3.6)	0.02
Repression & denial	42.8 (8.2)	42.6 (8.5)	43.3 (7.1)	ns
Distress endurance	48.1 (7.4)	47.5 (7.3)	50.1 (7.8)	0.04
Negative affect (DS14)	15.4 (7.7)	16.1 (7.5)	13.2 (8.0)	0.02
Avoidant coping (Brief COPE)	20.9 (4.4)	21.0 (4.4)	20.7 (2.2)	ns
Non-avoidant coping (Brief COPE)	50.7 (7.6)	50.6 (7.9)	50.9 (6.4)	ns
Perceived stress (PSS)	22.1 (6.7)	22.4 (7.1)	21.1 (5.2)	ns

The correlation coefficients for the relationships between the various inventories and subscales are shown in Table 2. Overall, scores on the AAQ-II had a strong positive relationship with total MEAQ scores which is consistent with prior findings (Gamez et al., 2011). More specifically, AAQ-II scores had moderate positive relationships with all MEAQ subscales with the exception of distress endurance which had a moderate negative relationship. MEAQ total scores had a strong positive relationship to the MEAQ subscales for behavioral avoidance, distress aversion, distraction and suppression, and repression and denial, but only a moderate positive relationship to procrastination, and a moderate negative relationship with distress endurance.

**Table 2:** Relationships between inventory scores and subscales.

	AAQ-II	MEAQ	Behavioral avoidance	Distress aversion	Procrastination	Distraction and suppression	Repression and denial	Distress endurance	Negative affect	Avoidance coping	Non-avoidance coping	Perceived stress
AAQ-II	–											
MEAQ	0.64**	–										
Behavioral Avoidance	0.44**	0.87**	–									
Distress Aversion	0.45**	0.82**	0.71**	–								
Procrastination	0.36**	0.38**	0.19*	0.12	–							
Distraction & Suppression	0.45**	0.72**	0.61**	0.59**	0.23*	–						
Repression & Denial	0.57**	0.82**	0.64**	0.55**	0.51**	0.54**	–					
Distress Endurance	−0.36**	−0.40**	−0.28**	−0.17*	0.15*	−0.01	−0.11	–				
Negative Affect (DS14)	0.66**	0.50**	0.37**	0.30**	0.33**	0.20*	0.46**	−0.39**	–			
Avoidance Coping (Brief COPE)	0.56**	0.50**	0.33**	0.30**	0.33**	0.30**	0.51**	−0.28**	0.54**	–		
Non-Avoidance Coping (Brief COPE)	−0.11	−0.13	0.08	0.01	0.05	−0.11	0.42**	−0.13	−0.13	0.02	–	
Perceived Stress (PSS)	0.48**	0.40**	0.26*	0.28**	0.33**	0.19*	0.37**	−0.23*	0.57**	0.41**	−0.07	–

**Notes:**

* *p* < 0.05.

** *p* < 0.001.

Another issue in the literature concerns the AAQ-II and MEAQ and their relationship to NA. As hypothesized, the AAQ-II had a strong positive relationship with the NA subscale of the DS-14 while the MEAQ had a moderate but significantly weaker relationship to NA than the AAQ-II (Fisher’s z transformation *z* = 2.42, *p* < 0.01). More specifically, the relationships of NA to the MEAQ subscales varied from a moderate positive relationship for repression and denial, distress aversion, procrastination, behavioral avoidance, to a weak positive relationship with distraction & suppression, to a moderate negative relationship for distress endurance.

Experiential avoidance as measured by the AAQ-II and the MEAQ total scores had a moderate positive relationship with an avoidance aggregate score consisting of behavioral disengagement, self-distraction, substance use, denial and self-blame scales from the Brief COPE. Specifically, AAQ-II scores had a weak to moderate positive relationship with the individual Brief COPE scales of behavioral disengagement (*r* = 0.39, *p* < 0.001), denial (*r* = 0.21, *p* < 0.05), substance use (*r* = 0.25, *p* < 0.001), and self-blame (*r* = 0.64, *p* < 0.001) with the exception of self-distraction which had a non-significant relationship (*r* = 0.14, *p* > 0.07). MEAQ total scores also had a weak to moderate positive relationship with all individual Brief COPE scales deemed avoidant including behavioral disengagement (*r* = 0.39, *p* < 0.001), denial (*r* = 0.34, *p* < 0.001), self-blame (*r* = 0.36, *p* < 0.001), self-distraction (*r* = 0.15, p < 0.05). and substance use (*r* = 0.26, *p* < 0.001). In addition to the avoidant coping styles, venting was positively correlated with MEAQ total score (*r* = 0.19, *p* < 0.05). In addition, all MEAQ subscale scores had a significant positive relationship to the avoidant aggregate score from the Brief COPE with the exception of distress endurance which had a weak negative relationship. The non-avoidant aggregate score from the Brief COPE which consists of acceptance, active coping, emotional support, humor, instrumental support, planning, positive reframing, religion, and venting had non-significant relationships with the AAQ-II and MEAQ total scores as well as all of the MEAQ subscale scores

The combined relationships of NA, avoidance coping on AAQ-II and MEAQ total scores were analyzed with stepwise linear regression which revealed that AAQ-II scores could be best predicted by a model including NA, avoidance coping, and MEAQ total scores (*R*^2^ = 0.574, *F*(3,199) = 90.4, *p* < 0.001). Stepwise linear regression also revealed MEAQ total scores could be best predicted by a model including avoidance coping and AAQ-II scores (*R*^2^ = 0.432, *F*(2,199) = 76.6, *p* < 0.001), but not NA.

Perceived stress had a moderate positive relationship to EA as measured by scores on both the AAQ II and the MEAQ. PSS scores had a weak to moderate positive relationship with scores on all MEAQ subscales (r’s ranged from 0.19 to 0.57) with the exception of distress endurance which had a weak negative relationship. Perceived stress also had a moderate positive relationship with avoidant coping styles from the Brief COPE and a moderate positive relationship with NA as measured by the DS-14. In addition, stepwise linear regression revealed that perceived stress could be best predicted by a model including NA and AAQ-II scores, but not MEAQ total scores (*R*^2^ = 0.34, *F*(2,199) = 51.37, *p* < 0.001).

## Discussion

The current study explored several open issues concerning the two most common measures of EA, the AAQ-II and the MEAQ. The current results confirmed only a moderate positive relationship between scores on the AAQ-II and the MEAQ which fits with previous conclusions that these EA measures do not assess the same construct (Lewis & Naugle, 2017; Rochefort, Baldwin & Chmielewski, 2018). Furthermore, the AAQ-II had moderate positive relationships with all MEAQ subscales with the exception of distress endurance which had a moderate negative relationship. This finding is consistent with Gamez et al. (2011) which indicated that distress endurance was consistently less related to the other MEAQ subscales and was the least representative measure of general EA in clinical and non-clinical populations. However, in subsequent reports, the relationship of distress endurance to other measures of EA has been inconsistent. Sahdra et al. (2016) found that distress endurance had weak to moderate negative relationships with the other MEAQ subscales with the exception of distraction and suppression which had a moderate positive relationship. However, Conley et al. (2019) reported that distress endurance had a strong positive relationship to the MEAQ total score and moderate positive relationships with the other MEAQ subscales. Based on these inconsistencies, further exploration of the relationship of distress endurance to EA is needed.

Acceptance and Action Questionnaire-II scores had a stronger relationship to NA than did MEAQ scores which fits with prior findings (Rochefort, Baldwin & Chmielewski, 2018) and supports the previous concerns that the AAQ-II cannot discriminate between EA and NA (Gamez et al., 2011; Wolgast, 2014; Rochefort, Baldwin & Chmielewski, 2018). This finding was further supported by the linear regression results which indicated that AAQ-II scores, but not MEAQ total scores, were predicted by a model that includes NA. In addition, the relationship of NA to the individual MEAQ subscales varied from moderate positive relationships with behavioral avoidance, distress aversion, procrastination and repression and denial to a weak positive relationship with distraction and suppression, to a moderate negative relationship with distress endurance. This finding is consistent with prior results of Buckner et al. (2014) which found that distress endurance has a weak negative relationship to negative affectivity.

Scores on the AAQ-II had a moderate positive relationship with an aggregate avoidance score from the Brief COPE which fits with prior findings (Karekla & Panayiotou, 2011). More specifically, AAQ-II scores were only weakly related to denial, substance use, self-blame but not behavioral disengagement or self-distraction. The current study extended this work to include the avoidant coping aggregate score from the Brief COPE which had a moderate positive relationship with MEAQ total scores as well as all of the MEAQ subscales with the exception of distress endurance which had a weak negative relationship. The current findings further our understanding of how the various dimensions of EA fit with avoidant coping styles.

The AAQ-II has also been criticized for not making a clear enough distinction between EA and perceived stress or psychological distress (Chawla & Ostafin, 2007; Batten, Follette & Aban, 2002; Marx & Sloan, 2005; Karekla & Panayiotou, 2011; Kashdan & Kane, 2011; Bardeen, Fergus & Orcutt, 2013). In the current study, AAQ-II scores had a stronger positive relationship with perceived stress than MEAQ total scores. This conclusion is also supported by the current linear regression results in which it was revealed that perceived stress could be best predicted by a model including NA and AAQ-II scores, but not MEAQ total scores. The current study also found a moderate positive relationship between perceived stress and negative affect which is consistent with a strong positive relationship as reported by Polman, Borkoles & Nicholls (2010).

All MEAQ subscales had weak to moderate positive relationships with perceived stress with the exception of distress endurance which had a moderate negative relationship to perceived stress. Buckner et al. (2014) found that distress endurance has a moderate negative relationship to the MEAQ total score and procrastination as well as a weak negative relationship to social anxiety. Sahdra et al. (2016) concluded that while distress endurance failed to account for unique variance of negative outcomes such as general mental distress, it did seem to be the most important of the MEAQ subscales in explaining unique variance in positive outcomes such as well-being and life satisfaction. However, Conley et al. (2019) reported that anticipatory anxiety had a non-significant relationship with distress endurance. Thus, the current findings align with findings that distress endurance is not related to negative outcomes such as perceived stress, social anxiety or anticipatory anxiety. Overall, the current findings support the relationship of EA as measured by the AAQ-II, but not the MEAQ, with perceived stress.

There was a significant gender effect for the AAQ-II such that females had higher scores than males which is consistent with the findings of Panayiotou, Karekla & Leonidou (2017) but not Bond et al. (2011). There was no significant gender effect for the MEAQ total scores, but analyses of MEAQ subscales revealed that females had higher distraction & suppression scores than males and males had higher distress endurance scores than females. There were no significant gender effects for avoidant or non-avoidant coping styles on the Brief COPE which fits with the findings of Allen & Myers (2019) but differs from previous findings that females are more likely than males to utilize avoidance and emotion-focused coping (Tamres, Janicki & Helgeson, 2002; Matud, 2004; Eaton & Bradley, 2008). There was a gender effect such that females had higher NA than males in the current study which supports the findings of Allen et al. (2018). The current finding of no gender effect for perceived stress in females is in contrast with prior findings (Karekla & Panayiotou, 2011). Overall, the current findings suggest the importance of including gender in future studies of avoidance.

## Limitations and future work

The current study has limitations and shortcomings which can be addressed in future work. The current work included a non-clinical sample of presumably healthy undergraduates that consisted of mainly female Caucasians from one university. There were no clinical assessments or measurements of anxiety beyond perceived stress. Future work examining the relationship between EA and other avoidant measures should include clinical populations especially those with post-traumatic stress disorder and anxiety disorders.

## Conclusions

Based on prior work and results of the current experiments, it can be concluded that the AAQ-II is not measuring EA in the same manner as the MEAQ. Another difference between the two measures of EA is that while the AAQ-II is limited as a unitary measure of EA the MEAQ may contain too many dimensions such as distress endurance which had a negative relationship to the AAQ-II or other avoidant constructs. MEAQ is also less related to NA, avoidant coping styles, and perceived stress than the AAQ-II. However, further refinement of MEAQ is still needed. Specifically, the idea that distress endurance is an outlier from the other dimensions of EA as well as related constructs such as negative affectivity and perceived stress should be explored. Given these uncertainties as to what the two most commons measures of EA actually measure, any conclusions on EA based on the AAQ-II or MEAQ should be cautious. Overall, the current study supported prior findings and adds some missing details on how EA, specifically as measured by MEAQ subscales, fits with avoidant constructs such as negative affect, avoidance coping, and perceived stress.

## Supplemental Information

10.7717/peerj.11033/supp-1 Supplemental Information 1 Item scores for the AAQ-II, MEAQ, NA measure, Brief COPE and PSS including code.

## Additional Information and Declarations

### Data Availability

The following information was supplied regarding data availability: Raw data are available as a Supplemental File.
